# TCTEX1D1 is a genetic modifier of disease progression in Duchenne muscular dystrophy

**DOI:** 10.1038/s41431-019-0563-6

**Published:** 2020-01-02

**Authors:** Pietro Spitali, Irina Zaharieva, Stefan Bohringer, Monika Hiller, Amina Chaouch, Andreas Roos, Chiara Scotton, Mireille Claustres, Luca Bello, Craig M. McDonald, Eric P. Hoffman, CINRG Investigators, Alberto Dubrovsky, Andrew Kornberg, Kathryn North, Monique Ryan, Richard Webster, W. Douglas Biggar, Laura C. McAdam, Jean K. Mah, Hanna Kolski, V. Vishwanathan, S. Chidambaranathan, Yoram Nevo, Ksenija Gorni, Jose Carlo, Mar Tulinius, Timothy Lotze, Tulio E. Bertorini, John W. Day, Peter Karachunski, Paula R. Clemens, Hoda Abdel-Hamid, Jean Teasley, Nancy Kuntz, Sherilyn Driscoll, John B. Bodensteiner, Anne M. Connolly, Alan Pestronk, R. T. Abresch, Erik K. Henricson, Nanette C. Joyce, Craig M. McDonald, Avital Cnaan, Heather Gordish-Dressmsn, Lauren P. Morgenroth, Robert Leshner, Carolina Tesi-Rocha, Mathula Thangarajh, Tina Duong, Zaida Koeks, H. Eka Suchiman, Sebahattin Cirak, Mariacristina Scoto, Mojgan Reza, Peter A. C. ‘t Hoen, Erik H. Niks, Sylvie Tuffery-Giraud, Hanns Lochmüller, Alessandra Ferlini, Francesco Muntoni, Annemieke Aartsma-Rus

**Affiliations:** 1 Department of Human Genetics, Leiden University Medical Center, Leiden, The Netherlands; 2 Dubowitz Neuromuscular Centre, University College London Great Ormond Street Institute of Child Health, London, UK; 3 Department of Medical Statistics, Leiden University Medical Center, Leiden, The Netherlands; 4 John Walton Muscular Dystrophy Research Centre, Newcastle University, Newcastle upon Tyne, UK; 5 Greater Manchester Neuroscience Centre, Salford Royal Foundation Trust, Salford, UK; 6 Department of Medical Sciences, Section of Microbiology and Medical Genetics, University of Ferrara, Ferrara, Italy; 7 Laboratory of Genetics of Rare Diseases (LGMR - EA7402), University of Montpellier, Montpellier, France; 8 Center for Genetic Medicine Research, Children’s National Medical Center, Washington, DC USA; 9 Department of Neuroscience, University of Padova, Padova, Italy; 10 University of California Davis Medical Center, Sacramento, CA USA; 11 Department of Neurology, Leiden University Medical Center, Leiden, The Netherlands; 12 Department of Molecular Epidemiology, Leiden University Medical Center, Leiden, The Netherlands; 13 Department of Pediatrics, University Hospital Cologne, Cologne, Germany; 14 Center for Molecular Medicine Cologne (CMMC), University of Cologne, Cologne, Germany; 15 Division of Neurology, Department of Medicine, The Ottawa Hospital, Ottawa, Canada; 16 Brain and Mind Research Institute, University of Ottawa, Ottawa, Canada; 17 National Institute for Health Research, Great Ormond Street Institute of Child Health Biomedical Research Centre, University College London, London, UK

**Keywords:** Prognostic markers, Genome-wide association studies, Neuromuscular disease

## Abstract

Duchenne muscular dystrophy (DMD) is caused by pathogenic variants in the *DMD* gene leading to the lack of dystrophin. Variability in the disease course suggests that other factors influence disease progression. With this study we aimed to identify genetic factors that may account for some of the variability in the clinical presentation. We compared whole-exome sequencing (WES) data in 27 DMD patients with extreme phenotypes to identify candidate variants that could affect disease progression. Validation of the candidate SNPs was performed in two independent cohorts including 301 (BIO-NMD cohort) and 109 (CINRG cohort of European ancestry) DMD patients, respectively. Variants in the Tctex1 domain containing 1 (*TCTEX1D1)* gene on chromosome 1 were associated with age of ambulation loss. The minor alleles of two independent variants, known to affect *TCTEX1D1* coding sequence and induce skipping of its exon 4, were associated with earlier loss of ambulation. Our data show that disease progression of DMD is affected by a new locus on chromosome 1 and demonstrate the possibility to identify genetic modifiers in rare diseases by studying WES data in patients with extreme phenotypes followed by multiple layers of validation.

## Introduction

Duchenne muscular dystrophy (DMD) is the most frequent childhood onset muscular dystrophy with an incidence of 1 in 5000 newborn males [1]. DMD is characterised by progressive loss of muscle mass and function [2], with most patients dying prematurely between the 2nd and 4th decade of life [3]. DMD is caused by pathogenic variants in the *DMD* gene, most commonly out-of-frame deletions, either de novo or X-linked recessively inherited, that result in the absence of the gene product called dystrophin [4, 5]. DMD natural disease course is well-documented in literature with disease milestones such as loss of ambulation, dilated cardiomyopathy and respiratory complications, which significantly affect the survival of affected individuals [6, 7]. Recent re-evaluation of disease natural history with several quantitative tests [8–14] enabled the identification of patients progressing faster than others [11, 13–16]. Several studies have been carried out to identify the genetic factors responsible for the observed differences. This work revealed that both the deletion site (*cis* effect) as well as variants in other genes (*trans* effect) could influence the disease course. Specifically, it was observed that patients carrying out-of-frame deletions flanking exon 44 experience a longer ambulatory phase compared with patients carrying other out-of-frame deletions [15]. The milder phenotype of exon 44 skippable patients is attributed to the higher residual dystrophin production of these patients compared with other DMD genotypes [15, 17]. Other genes acting in *trans* were recently described as genetic modifiers of DMD, namely *SPP1* (chromosome 4), *LTBP4* (chromosome 19) and *CD40* (chromosome 20) [18–20]. The first variant to be described is a non-coding SNP located 5 nucleotides upstream the transcription start site of the *SPP1* gene encoding osteopontin [16, 18, 19, 21, 22]. *LTBP4* was first identified in a murine model of muscular dystrophy and then validated in several independent DMD cohorts [16, 19, 22, 23]. *CD40* was identified as a modifier locus by genotyping functionally relevant SNPs with an exome chip, and selecting candidate genes with a biological role in pro-inflammatory and pro-fibrotic pathways [20]. None of these modifiers has been identified by conducting a genome-wide association study (GWAS), due to the low prevalence of the disease, which precluded the design of a properly powered discovery study. Recently a GWAS performed in 253 patients with dystrophinopathy identified *THBS1* (chromosome 15) as a potent DMD modifier, showing that genome-wide significance is achievable with relatively low numbers [24]. To overcome the power limitation we took a different approach based on deep genotyping of patients with extremely different clinical presentations, followed by validation in two large multicentre cohorts. To identify candidate genetic modifying variants, we performed whole-exome sequencing (WES) of patients affected by DMD with extreme phenotypes. We then validated the candidate variants in a multi-centre European cohort (Bio-NMD cohort) composed of 301 patients with DMD. The analysis has enabled us to identify 11 SNPs associated with age of ambulation loss. To further validate the identified SNPs, we explored the association with age of ambulation loss in a second independent American DMD cohort, which confirmed our findings. This enabled us to identify a locus on chromosome 1 influencing disease progression in patients with DMD.

## Materials and methods

### Study participants

#### Discovery cohorts

This study comprised a discovery phase, and a validation phase.

Comparisons of patients with extreme phenotypes were performed during the discovery phase, including patients characterised by:

- early ambulation loss (before 8.5 years of age, *n* = 5) in contrast to patients who retained the ability to walk longer than average (loss of ambulation after 12 years of age, *n* = 5) (ambulation group),
- early and severe cardiomyopathy, defined as an ejection fraction <40% or fraction shortening <15% (before 13 years of age, *n* = 6) in contrast to patients who were alive at the age of 28 with no detectable or only mild heart involvement, defined as an ejection fraction between 45 and 54% or fraction shortening between 20 and 27% (*n* = 11) (cardiomyopathy group)

Table 1 shows the characteristics of patients involved in both studies. Pathogenic variants have been submitted to the Leiden Open Variation Database (LOVD) [25] at https://databases.lovd.nl/shared/individuals/ (ID 00265240, 00265263-00265264, 00265266-00265279, 00265281-00265285, 00265287 and 00265396).

**Table 1:** Characteristics of the extreme DMD phenotype groups.

ID	Variant description	Exons Involved	Phenotype group	Age LoA (years)	Steroid treatment before LoA	Age at last visit (years)	Age of onset of cardiomyopathy (years)
Extreme phenotype cohort: age of loss of ambulation							
ES0SN	NC_000023.11(NM_004006.2):c.(6912 + 1_6913 − 1)_(7309 + 1_7310 − 1)del	48–50	ELoA	6	no	na	na
ES065	NC_000023.11(NM_004006.2):c.2098C>T	17	ELoA	7	Yes	na	na
ES005	NC_000023.11(NM_004006.2):c.(649 + 1_650 − 1)_(1482 + 1_1483 − 1)del	8–12	ELoA	7.5	Yes	na	na
ES074	NC_000023.11(NM_004006.2):c.(960 + 1_961 − 1)_(1331 + 1_1332 + 1)del	10–11	ELoA	8	Yes	na	na
ES067	NC_000023.11(NM_004006.2):c.(6614 + 1_6615 − 1)_(7542 + 1_7543 − 1)del	46–51	ELoA	8.5	Yes	na	na
ES028	NC_000023.11(NM_004006.2):c.(6614 + 1_6615 − 1)_(7200 + 1_7201 − 1)del	46–49	LLoA	12	Yes	na	na
ES050	NC_000023.11(NM_004006.2):c.(6438 + 1_6439 − 1)_(6614 + 1_6615 − 1)del	45	LLoA	12	Yes	na	na
ES075	NC_000023.11(NM_004006.2):c.(7309 + 1_7310 − 1)_(7542 + 1_7543 − 1)del	51	LLoA	12	Not known	na	na
ES045	NC_000023.11(NM_004006.2):c.(264 + 1_265 − 1)_(649 + 1_650 − 1)del	5–7	LLoA	15	Yes	na	na
ES0SW	NC_000023.11(NM_004006.2):c.(5922 + 1_5923 − 1)_(6290 + 1_6291 − 1)del	42–43	LLoA	Ambulant at 14	Yes	na	na
Extreme phenotype cohort: early cardiomyopathy/long survivor							
SS2	NC_000023.11(NM_004006.2):c.1886C>G	16	LS	na	na	32	Not documented
SS3	NC_000023.11(NM_004006.2):c.6553_6553delT	45	LS	na	na	30	Not documented
SS4	NC_000023.11(NM_004006.2):c.(7309 + 1_7310 − 1)_(7542 + 1_7543 − 1)del	51	LS	na	na	33	Not documented
SS6	NC_000023.11(NM_004006.2):c.(6912 + 1_6913 − 1)_(7309 + 1_7310 − 1)del	48–50	LS	na	na	38	Mild ventricular dysfunction at 35
SS7	NC_000023.11(NM_004006.2):c.(6438 + 1_6439 − 1)_(6762 + 1_6763 − 1)del	45–46	LS	na	na	31	Not documented
SS9	NC_000023.11(NM_004006.2):c.(7309 + 1_7310 − 1)_(7542 + 1_7543 − 1)del	51	LS	na	na	28	22
SS11	NC_000023.11(NM_004006.2):c.(93 + 1_94 − 1)_(649 + 1_650 − 1)dup	3–7	LS	na	na	31	Normal evaluation at 28
SS12	NC_000023.11(NM_004006.2):c.(6614 + 1_6615 − 1)_(7309 + 1_7310 − 1)del	46–50	LS	na	na	31	Not documented
SS13	NC_000023.11(NM_004006.2):c.(6438 + 1_6439 − 1)_(6614 + 1_6615 − 1)del	45	LS	na	na	31	Mild ventricular dysfunction at 28
SS17	NC_000023.11(NM_004006.2):c.10265dupC	72	LS	na	na	39	No cardiac impairment
SS18	NC_000023.11(NM_004006.2):c.(31 + 1_32 − 1)_(93 + 1_94 − 1)dup	2	LS	na	na	32	Not documented
SS1	NC_000023.11(NM_004006.2):c.(8217 + 1_8218 − 1)_(8547 + 1_8548 − 1)dup	56–57	ECM	na	na	na	5
SS8	NC_000023.11(NM_004006.2):c.(7098 + 1_7099 − 1)_(7309 + 1_7310 − 1)del	49–50	ECM	na	na	na	Cardiorespiratory arrest at 13
SS10	NC_000023.11(NM_004006.2):c.(6290 + 1_6291 − 1)_(6912 + 1_6913 − 1)del	44–47	ECM	na	na	na	11
SS14	NC_000023.11(NM_004006.2):c.(6117 + 1_6118 − 1)_(7542 + 1_7543 − 1)del	43–51	ECM	na	na	na	11
SS15	NC_000023.11(NM_004006.2):c.(264 + 1_265 − 1)_(4071 + 1_4072 − 1)del	5–29	ECM	na	na	na	11
SS16	NC_000023.11(NM_004006.2):c.(264 + 1_265 − 1)_(2380 + 1_2381 − 1)dup	5–19	ECM	na	na	na	8

Pathogenic variant, age of loss of ambulation, corticosteroid treatment, age of the last clinical assessment and onset of cardiomyopathy. Pathogenic variants are now described using the chromosome position and annotated to the main isoform (Dp427m) with reference sequence NM_004006.2. Exon numbering is based on exons annotated in the reference sequence NM_004006.2. Exons were numbered sequentially staring with exon 1 mapping to nucleotides 1–275. Numbering of downstream exons follows sequentially with e.g. exon 2 directly following exon 1 and being composed of nucleotides 276 up to 337 of NM_004006.2

*LoA* loss of ambulation, *ELoA* early loss of ambulation, *LLoA* late loss of ambulation, *LS* long survivor, *ECM* early cardiomyopathy, *na* not applicable

#### Validation cohorts

In the validation phase, two independent cohorts were studied. The first validation cohort was a multi-centre European cohort composed of 301 patients with DMD hereafter referred to as Bio-NMD cohort. The second validation cohort was composed of 109 individuals with DMD of European or European-American ancestry (selected by multidimensional scaling of genome-wide SNP genotyping data as previously described [16, 20]) participating in a DMD natural history study [8] (CINRG cohort). Table 2 shows the characteristics of the two validation cohorts.

**Table 2:** Characteristics of the validation cohorts.

	Age				Ambulation				Corticosteroids					
	Mean	Min	Med	Max	Ambulant		Non ambulant		Not treated		Treated		Unknown	
					*N*	Row %	*N*	Row %	N	Row %	*N*	Row %	Count	Row %
BIO-NMD cohort														
Newcastle	10.11	2.9	10	17.1	30	39.50%	46	60.50%	35	46.10%	34	44.70%	7	9.20%
London	10.53	4	11	15	7	13.00%	47	87.00%	32	59.30%	21	38.90%	1	1.90%
Ferrara	9.86	3.8	10	17	0	0.00%	62	100.00%	30	48.40%	13	21.00%	19	30.60%
Montpellier	9.56	5.8	9	14	0	0.00%	42	100.00%	36	85.70%	2	4.80%	4	9.50%
Leiden	9.31	4	9	13	17	25.40%	50	74.60%	36	53.70%	27	40.30%	4	6.00%
CINRG cohort (European Ancestry sub-cohort)	12.53	3	11.4	25.7	40	36.70%	69	63.30%	22	20.20%	87	79.80%	0	0%

Details about the age, ambulation status and corticosteroid usage are provided for the BIO-NMD and CINRG validation cohorts

Local Research Ethics Committees of all participating institutions (Leiden University Medical Center, University College London, Newcastle University, University of Ferrara and University of Montpellier) approved the study prior to its start. Informed consent for anonymised use of patients’ data was obtained for all patients. All methods were performed in accordance with the relevant institutional and country guidelines and regulations.

##### Identification of candidate genetic modifiers

Samples included in the discovery cohorts were prepared for sequencing using Agilent Sure Select AllExon 50 Mb XT kit according to the manufacturer’s protocol.

The prepared exome libraries for the ambulation group were sequenced on Illumina GAIIx with 2 × 76 bp number of cycles (pipeline version OLB 1.8, Casava 1.7). The average coverage of the sequenced regions was 50×. The analysis of the sequencing reads was performed using General Application Pipeline for Second generation Sequencing (GAPSS3) developed by the Department of Human Genetics, LUMC, The Netherlands. GAPSS3 performs quality control of the input data, alignment of the improved data using Stampy [26] and variant calling through SAMtools [27].

The exome libraries for the cardiomyopathy group were sequenced on an Illumina HiSeq with 100 bp paired-end. The average coverage of the sequenced regions was 54×. The data analysis was performed in-house with alignment of the data using Novoalign (www.novocraft.com) and variant calling through SAMtools.

The functional annotation of the genetic variants for both studies was performed using ANNOVAR software tool [28].

Variants showing concordant genotypes were filtered out. The criteria for selecting a SNP as a candidate genetic modifier were: (1) exonic non-synonymous SNPs; (2) the minor allele should be found in only one group; (3) and it should be present in at least 50% of the cases in the specific group. All filtering steps were performed using ANNOVAR software tool.

The selected candidate genetic modifiers were subject to further technical validation using AmpliSeq Ion Torrent Technology (Life Technologies). Custom panels with primer pairs covering the selected SNPs were designed and analysed in the same DNA samples were used in the WES discovery step.

### Validation of candidate SNPs

The Sequenom (Sequenom Inc, San Diego, California, USA) MassARRAY platform was used to validate the genetic associations in the BIO-NMD cohort. Platform and software were used according to the manufacturer’s protocols. The protocol described by van den Bergen et al. [22] was used to assay all SNPs. Primers for multiplex genotyping assays were designed and sequences are available upon request. Genotyping of the CINRG cohort was performed using the Illumina Human Exome Chip previously reported [16, 20].

### Statistical analysis

Cox regression was used to identify association of SNPs with time to ambulation loss. An additive genetic model was used throughout to determine genotype effects. Corticosteroid use, cohort (Ferrara, Leiden, London, Montpellier and Newcastle) and the interaction between genotype and corticosteroid use were included in the analysis as covariates. Cox regression was also used to validate SNP associations in the CINRG cohort, with an additive genetic model and a covariate for corticosteroid treatment (at least 1 year of treatment while ambulatory). Global *P* values were calculated using the tail-strength method [29], the P-min and the SKAT [30] statistics. In short, the tail-strength measures how much *P* values in a set differ from the expected uniform distribution under the null hypothesis and sums up these differences into a single test statistic. The tail-strength is powerful when many small effects exist in the data [29]. The P-min test usually performs well when a few stronger signals are present in the data. Since SNPs are not independent, empirical *P* values were computed using permutations for the *tail strength* and *P-min* statistics. SNPs were permuted as a block. This keeps the relationship between genotypes intact as well as between the other covariates and outcome but breaks the relationship between genotypes and outcome. Individual tests were based on a cox-model (*coxph*) and 1 × 10^4^ permutations were used. Computations were parallelised using package *parallelise.dynamic* [31]. Global *P* values were computed using R version 3.2. The *SKAT* statistic does not assume independence of SNPs and was computed using *bioconductor* package *globaltest* [32] without permutations. We also computed the statistics *SKAT standardised* which is the SKAT statistics computed on standardised genotypes. Statistical analysis of genotype data for the top 10 SNPs in the CINRG cohort was performed using Cox regression of age at loss of ambulation with an additive SNP effect and a covariate for corticosteroid use (at least 1 year while ambulatory vs less or untreated). The interaction term between genotype and corticosteroid treatment was not included in the analysis of the CINRG cohort due to the low minor allele frequency of the ten variants selected for replication. Variants associated with loss of ambulation were submitted to the LOVD at https://databases.lovd.nl/shared/individuals/ (ID 00264110-00264115 and 00265391-00265395).

Bioinformatic analysis was performed by using the GeneTrail2 [33] and GTex [34] online tools. GeneTrail2 was used to test whether any of the known MEOX2 binders could be linked to the pathological pathways known to be altered in DMD. The list of MEOX2 binders (including experimental and predicted binding proteins) was obtained by Integrated Interaction Database [35] and used as input in GeneTrail2. Over-representation analysis using all supported human Uniprot IDs as background was performed. Only pathways obtained from Reactome were tested. A two-sided test followed by multiple testing correction by Benjamini and Hochberg false discovery rate (FDR) was performed and a FDR < 5% was considered significant. To assess whether the variants in the *TCTEX1D1* gene were previously associated with the expression of neighbouring genes in muscle tissue, the GTex database was consulted. The rs number of the two variants was submitted and the significant expression quantitative trait loci (eQTLs) for muscle and heart tissues are reported.

## Results

### Identification of candidate genetic modifiers

**Fig. 1:**
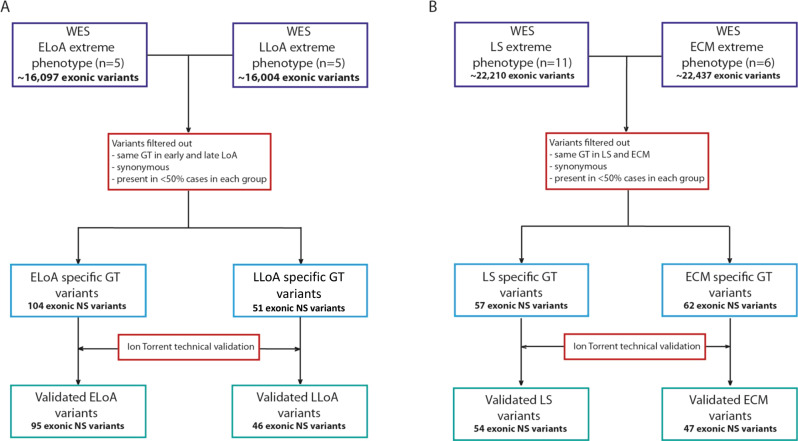
Workflow of genetic biomarker identification in DMD patients with extreme phenotype. **a** DMD patients with early loss of ambulation (before the age of 8.5 years) and late loss of ambulation (after 12 years). **b** DMD patients with late onset cardiomyopathy and patients with early onset of cardiomyopathy. WES whole-exome sequencing, LoA loss of ambulation, GT genotype, NS non-synonymous.

We performed WES in 27 patients with DMD with extreme phenotypes in order to identify candidate genetic modifiers. Subjects are described in Table 1. In the first comparison the SNPs genotypes in five patients characterised by early loss of ambulation (ELoA) were compared with the ones obtained in five patients with late loss of ambulation (LLoA) (Fig. 1a). About 16,000 exonic variants were analysed in each group. After filtering out the SNPs with concordant genotype between groups, intronic SNPs, synonymous SNPs and SNPs present in <3 cases per group, we were left with 104 SNPs associated with ELoA and 51 SNPs associated with LLoA. Samples resequencing by Ion Torrent confirmed the genotype of 95 ELoA SNPs and 46 LLoA SNPs.

In the second comparison, six patients with DMD with early cardiomyopathy (ECM) were compared with eleven patients with long survival and no substantial cardiomyopathy (LS) (Fig. 1b). More than 22,000 exonic variants were analysed in each group. After filtering out SNPs following the same criteria as above, 62 SNPs were associated with ECM and 57 SNPs with LS. Resequencing of DNA samples confirmed 47 ECM SNPs and 54 LS SNPs. All SNPs that passed the technical validation were selected for further validation.

### Validation of candidate SNPs

**Fig. 2:**
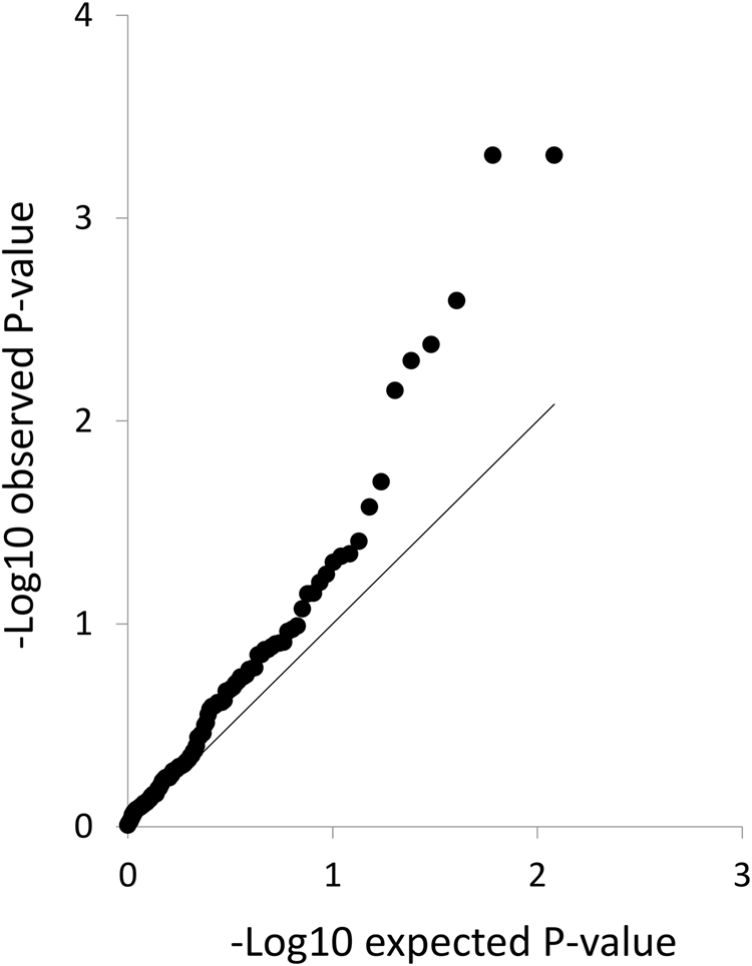
Q–Q plot showing the results obtained in the BIO-NMD validation cohort. The observed *P* values are lower compared with the expected ones showing an enrichment in the obtained distribution compared with the observed one.

A total of 242 SNPs entered the validation phase, 199 of which were successfully genotyped in the first validation cohort consisting of 301 patients with DMD (Bio-NMD cohort, described in the methods section and in Table 2). The remaining 43 SNPs were excluded as they did not fit in the plate design or due to the sequence context which did not allow genotyping of those positions. SNPs were excluded before data analysis for minor allele frequencies below 10%, for violation of the Hardy Weinberg equilibrium or for high genotype missingness (top 5% of SNPs with highest percentage of missing data). The remaining 121 SNPs provided in Supplementary Table 1 were included in the analysis. Under *P-min* model, there was no single SNP strongly associated with age of ambulation loss. However, the *tail strength*, *SKAT* model and *SKAT standardised* models showed significant association for 11 SNPs with age of ambulation loss (*P* < 0.05 for all three models). A summary of the results obtained is presented in Table 3. The Q–Q plot presented in Fig. 2 shows an enrichment of observed *P* values compared with the expected ones, which is to be expected in the validation phase where candidate genes are analysed. Of note, SNPs in complete linkage disequilibrium (LD) were identified at two different loci. Interestingly, for six SNPs a significant interaction with corticosteroid treatment was found (Table 3).

**Table 3:** Rs number, gene name, chromosome, discovery study and *P* value of the SNPs validated in Bio-NMD and CINRG cohorts.

*n*	SNP	Gene	Chr	Discovery study	Global *P* value BIO-NMD cohort	SNP main effect *P* value BIO-NMD	SNP and steroid interaction *P* value BIO-NMD	*P* value CINRG
1	rs1060575	*TCTEX1D1*	1	ECM vs LS	**0.004205996**	**0.000489**	**0.014307**	**0.032**
2	rs3816989	*TCTEX1D1*	1	ECM vs LS	**0.004205996**	**0.000489**	**0.014307**	**0.032**
3	rs566655	*LAMA1*	18	ELOA vs LLOA	**0.01300225**	**0.002543**	0.211529	NS
4	rs2074912	*PCDHGC5*	5	ELOA vs LLOA	**0.01365342**	**0.00503**	0.506818	NS
5	rs1058405	*ATF6*	1	ELOA vs LLOA	**0.014588146**	**0.004185**	0.309454	NS
6	rs3754689	*LCT*	2	ELOA vs LLOA	**0.014749404**	**0.00706**	**0.036407**	NS
7	rs2108485	*MTERFD2*	2	ECM vs LS	**0.022770651**	0.523446	0.066477	NS
8	rs2298831	*AMICA1*	11	ELOA vs LLOA	**0.041684306**	0.762777	**0.023477**	NS
9	rs10462020	*PER3*	1	ELOA vs LLOA	**0.043016839**	0.129351	**0.011065**	NS
10	rs10462021	*PER3*	1	ELOA vs LLOA	**0.04631428**	0.133637	**0.012061**	NS
11	rs12146487^a^	*PLCB3*	11	ECM vs LS	**0.049617866**	0.056927	0.785094	NS

^a^rs12146487 was not genotyped in the CINRG cohort and the given *P* value refers to SNP rs2244621 in the same region Significant *P* values are shown in bold

*NS* not significant

### External validation confirms an association with a locus on chromosome 1

**Fig. 3:**
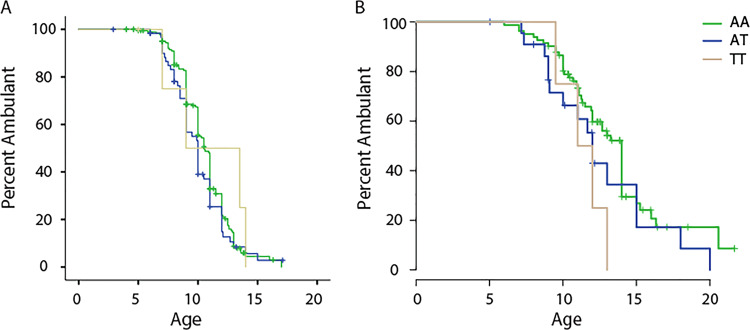
Kaplan–Meier curves showing the effect of SNPs in chromosome 1 on age of ambulation loss. **a** The effect of rs1060575 in the BIO-NMD validation cohort. **b** The SNP effects in the CINRG validation cohort. Censored patients are indicated by a cross on the lines.

To further validate the identified associations, we investigated the 11 SNPs confirmed in the Bio-NMD cohort in an independent cohort of 109 patients with DMD of European ancestry followed up in a multi-centre DMD natural history cohort (described in the methods section and in Table 2). Genotype data were already available for these patients since they were included in a recent exome chip association study involving patients with DMD [20]. Ten of eleven SNPs were present in the published dataset, while genotyping data were not available for SNP rs12146487 and therefore SNP rs2244621 in close proximity (419 bp) was considered instead. Analysis of the CINRG cohort showed that the two SNPs (rs1060575 and rs3816989) in LD at the *TCTEX1D1* locus were associated with age at loss of ambulation in the CINRG cohort (*P* = 0.032) (Fig. 3). Both SNPs have an effect on the transcript originating from this locus. SNP rs1060575 is an A to T transversion responsible for an aminoacidic change from glutamate to aspartate in exon 3 (https://www.ncbi.nlm.nih.gov/SNP/snp_ref.cgi?type=rs&rs=rs1060575), while rs3816989 is G to A transition affecting the donor splice site of exon 4 and inducing the skipping of exon 4 [36]. The minor alleles were associated with a faster disease progression as shown by the Kaplan–Meier curves (Fig. 3).

## Discussion

Patients affected by DMD experience a rather predictable disease course with known disease milestones. International collaborations and deep phenotyping have enabled to collect and analyse a large body of data that allowed the identification of different disease trajectories across patients [37, 38]. The identification of factors that affect disease progression is of interest as these factors may represent both prognostic biomarkers and potential therapeutic targets. Differences in disease progression have been connected to the deletion site within the *DMD* gene [15, 17] as well as to other factors such as disease modifying SNPs in *SPP1*, *LTBP4*, *CD40* and *THBS1* genes [18–20, 22, 24]. The rare disease nature of the disease has not allowed to properly power a GWA study in DMD. However the strong collaboration of international networks, as well as deep phenotyping of patients, has been the inspiration to perform an exome wide discovery study followed by multiple layers of validation. Indeed, we present here WES data of patients with DMD with extreme phenotypes based on the hypothesis that analysing homogeneous groups of patients with extreme phenotype would increase the chance to detect genetic modifiers in a small number of patients. A similar approach has been successfully used in patients affected by cystic fibrosis but never before in neuromuscular patients [39]. The discovery phase focused on two major variables responsible for DMD morbidity and mortality: skeletal muscle weakness (and loss of ambulation as its direct surrogate) and severity of cardiomyopathy. The cut-off values were chosen based on the personal experience of the paediatric neurologists participating to the BIO-NMD consortium, local clinical databases and natural history reports describing different DMD trajectories. Our recently concluded natural history studies further allowed us to refine the proposed categorisation based on estimates derived from the distribution of the events over the age in natural history studies [38]. We reached a consensus that losing ambulation before 8.5 years of age was a clinically meaningful change compared with after 12. Using this categorisation we were able to segregate patients belonging to different categories. Published estimates for loss of ambulation consistently report a mean age of 10 [22, 24, 37]. The most recent report showed a mean of 10.6 with standard deviation [SD] = 2.3 [24]. The 8.5 threshold is therefore about 1 SD below the mean. Comparable considerations have been used for the cardiomyopathy group. Comparison of extreme cases enabled to identify 242 candidate DMD modifying variants to be investigated further. A first layer of validation in a cohort of 301 patients with DMD with the whole spectrum of clinical severity, representing the largest European DMD cohort described so far, confirmed a significant association of 11 SNPs with the age of ambulation loss. Subsequent validation in the CINRG Duchenne Natural History study considering participants of European ancestry narrowed down the number of candidates to two SNPs (rs1060575 and rs3816989) in complete LD in the *TCTEX1D1* locus on chromosome 1. SNP rs1060575 is a missense SNP leading to a likely benign substitution (according to SIFT [40] and PolyPhen [41]) of glutamic acid with aspartic acid in exon 3, which is also observed in other species. The second SNP rs3816989 is located at position +1 of the donor splice site of exon 4 and it has been reported to lead to *TCTEX1D1* exon 4 skipping [36] reducing the resulting polypeptide chain from 179 to 72 amino acid residues. As yet the role of TCTEX1D1 is largely unknown, so it is uncertain how the lack of amino acids affects protein function. The gene is more highly expressed in brain according to the Expression Atlas (EMBL-EBI) [42], and it is expressed at lower level in skeletal muscle and heart. There is evidence for TCTEX1D1 protein interaction with MEOX2 (Mesenchyme Homeo Box 2) protein [43]. MEOX2 is expressed in skeletal muscle, it regulates muscle progenitor cells such as Pax3/7 positive cells and it is involved in limb myogenesis in vertebrates. MEOX2 null mice show hypertrophic, centrally nucleated fibres (normally found in *mdx* mice as a consequence of muscle regeneration) as well as a shift towards oxidative type I fibres and reduced myonuclear domains. It is over-expressed in muscle biopsies of patients with DMD as well as in patients affected by Emery–Dreifuss muscular dystrophy and juvenile dermatomyositis [44]. A pathway analysis performed with GeneTrail2 [33] including all known and potential MEOX2 binding partners (obtained from the Integrated Interaction Database [35]) revealed that MEOX2 interacting partners are involved in a number of pathways previously related to DMD pathophysiology such as NF-κB and SMAD signalling.

Both rs1060575 and rs3816989 variants were identified in the comparison between patients showing early signs of cardiomyopathy with long survivor patients, without significant cardiac involvement. Given that the discovery cohort was originally focused also on the cardiac phenotype; the association with loss of ambulation suggests that the identified gene and variants can have a role on both cardiac and skeletal muscles. To understand whether SNPs rs1060575 and rs3816989 drive the expression of the *TCTEX1D1* gene and other neighbouring genes, we searched for eQTLs in the GTEx database. Interestingly, the minor alleles of these variants are associated with the reduced expression of *TCTEX1D1* and an increased expression of the neighbouring gene *SGIP1* in both muscle and heart (Supplementary Fig. 1). Given that the minor allele was associated with a worse outcome in both discovery and validation cohorts, one could postulate that an increased expression of *SGIP1* and a decreased expression of *TCTEX1D1* could be deleterious for the cardiac phenotype in patients with DMD. Interestingly, a recent GWA meta-analysis for quantitative electrocardiography traits, showed that variants in the *TCTEX1D1/SGIP1* locus are associated with QT interval [45]. More research (e.g. conditional knock down of *TCTEX1D1* in skeletal and cardiac muscles in animal models or study the different *TCTEX1D1* proteoforms in patients’ derived cells) will be needed to provide solid mechanistic explanation of the role of these variants in DMD disease progression.

The comparison between the ELOA and LLOA did not lead to the identification of other variants. One possible cause for this is the selection of patients involved in the LLOA discovery cohort in which two patients carried out-of-frame deletions that can be reframed by exon 44 skipping. While it is now known that patients with this genomic characteristic experience a somewhat milder disease progression [15, 17], this was not known when the study was designed. An improved patients selection could have made the comparison more informative for the identification of other variants possibly acting *in trans*.

Our study is the first to identify modifiers associated with cardiomyopathy severity in DMD; the obtained data will now support the interpretation of studies on cardiac protection.

To the best of our knowledge, our work is the first in which exome sequencing was used to identify genetic modifiers in patients affected by DMD. In view of the relative rarity of DMD and the strategy to concentrate on the extreme phenotypes, the study was underpowered. However, multiple layers of subsequent validation enabled us to exclude false positive signals and identify *TCTEX1D1* as modifier of DMD disease progression. The success of this approach could be beneficial also for other rare diseases where the small cohorts size does not enable a classical GWAS design.

## Supplementary information

Supplementary Table 1

Supplementary Figure 1

Supplemental Material Legends

## References

[1] Norwood FLM, Harling C, Chinnery PF, Eagle M, Bushby K, Straub V (2009). Prevalence of genetic muscle disease in Northern England: in-depth analysis of a muscle clinic population. Brain.

[2] Straub V, Balabanov P, Bushby K, Ensini M, Goemans N, De Luca A (2016). Stakeholder cooperation to overcome challenges in orphan medicine development: the example of Duchenne muscular dystrophy. Lancet Neurol.

[3] Bushby K, Finkel R, Birnkrant DJ, Case LE, Clemens PR, Cripe L (2010). Diagnosis and management of Duchenne muscular dystrophy, part 1: diagnosis, and pharmacological and psychosocial management. Lancet Neurol.

[4] Ellis JA, Vroom E, Muntoni F (2013). 195th ENMC International Workshop: newborn screening for Duchenne muscular dystrophy 14–16th December, 2012, Naarden, The Netherlands. Neuromuscul Disord.

[5] Aartsma-Rus A, Ginjaar IB, Bushby K (2016). The importance of genetic diagnosis for Duchenne muscular dystrophy. J Med Genet.

[6] Desguerre I, Christov C, Mayer M, Zeller R, Becane H-M, Bastuji-Garin S (2009). Clinical heterogeneity of duchenne muscular dystrophy (DMD): definition of sub-phenotypes and predictive criteria by long-term follow-up. PLoS One.

[7] Magri F, Govoni A, D’Angelo MG, Del Bo R, Ghezzi S, Sandra G (2011). Genotype and phenotype characterization in a large dystrophinopathic cohort with extended follow-up. J Neurol.

[8] Henricson EK, Abresch RT, Cnaan A, Hu F, Duong T, Arrieta A (2013). The cooperative international neuromuscular research group Duchenne natural history study: glucocorticoid treatment preserves clinically meaningful functional milestones and reduces rate of disease progression as measured by manual muscle testing and other. Muscle Nerve.

[9] Henricson E, Abresch R, Han JJ, Nicorici A, Goude Keller E, de Bie E et al. The 6-minute walk test and person-reported outcomes in boys with duchenne muscular dystrophy and typically developing controls: longitudinal comparisons and clinically-meaningful changes over one year. PLoS Curr. 2013. 10.1371/currents.md.9e17658b007eb79fcd6f723089f79e06. 10.1371/currents.md.9e17658b007eb79fcd6f723089f79e06 PMC3712467 23867975

[10] Mcdonald CM, Henricson EK, Abresch RT, Florence JM, Eagle M, Gappmaier E (2013). THE 6-minute walk test and other endpoints in Duchenne muscular dystrophy: Longitudinal natural history observations over 48 weeks from a multicenter study. Muscle Nerve.

[11] McDonald CM, Henricson EK, Abresch RT, Florence J, Eagle M, Gappmaier E (2013). The 6-minute walk test and other clinical endpoints in duchenne muscular dystrophy: reliability, concurrent validity, and minimal clinically important differences from a multicenter study. Muscle Nerve.

[12] Mazzone ES, Messina S, Vasco G, Main M, Eagle M, D’Amico A (2009). Reliability of the north star ambulatory assessment in a multicentric setting. Neuromuscul Disord.

[13] Mazzone E, Vasco G, Sormani MP, Torrente Y, Berardinelli A, Messina S (2011). Functional changes in Duchenne muscular dystrophy: a 12-month longitudinal cohort study. Neurology.

[14] Ricotti V, Ridout DA, Pane M, Main M, Mayhew A, Mercuri E et al. The NorthStar Ambulatory Assessment in Duchenne muscular dystrophy: considerations for the design of clinical trials. J Neurol Neurosurg Psychiatry. 2015. 10.1136/jnnp-2014-309405. 10.1136/jnnp-2014-309405 PMC4752678 25733532

[15] van den Bergen JC, Ginjaar HB, Niks EH, Aartsma-Rus AVJJGM, van den Bergen J, Ginjaar H (2014). Prolonged ambulation in Duchenne patients with a mutation amenable to exon 44 skipping. J Neuromuscul Dis.

[16] Bello L, Kesari A, Gordish-Dressman H, Cnaan A, Morgenroth LP, Punetha J (2015). Genetic modifiers of ambulation in the cooperative international neuromuscular research group Duchenne natural history study. Ann Neurol.

[17] Anthony K, Arechavala-Gomeza V, Ricotti V, Torelli S, Feng L, Janghra N (2014). Biochemical characterization of patients with in-frame or out-of-frame DMD deletions pertinent to exon 44 or 45 skipping. JAMA Neurol.

[18] Pegoraro E, Hoffman EP, Piva L, Gavassini BF, Cagnin S, Ermani M (2011). SPP1 genotype is a determinant of disease severity in Duchenne muscular dystrophy. Neurology.

[19] Flanigan KM, Ceco E, Lamar KM, Kaminoh Y, Dunn DM, Mendell JR (2013). LTBP4 genotype predicts age of ambulatory loss in duchenne muscular dystrophy. Ann Neurol.

[20] Bello L, Flanigan KM, Weiss RB, Spitali P, Aartsma-Rus A, United Dystrophinopathy Project (2016). Association study of exon variants in the NF-κB and TGFβ pathways identifies CD40 as a modifier of duchenne muscular dystrophy. Am J Hum Genet.

[21] Bello L, Piva L, Barp A, Taglia A, Picillo E, Vasco G (2012). Importance of SPP1 genotype as a covariate in clinical trials in Duchenne muscular dystrophy. Neurology.

[22] van den Bergen JC, Hiller M, Böhringer S, Vijfhuizen L, Ginjaar HB, Chaouch A et al. Validation of genetic modifiers for Duchenne muscular dystrophy: a multicentre study assessing SPP1 and LTBP4 variants. J Neurol Neurosurg Psychiatry. 2015;86:1060–5. 10.1136/jnnp-2014-308409 PMC4602257 25476005

[23] Lamar K, McNally EM (2014). Genetic modifiers for neuromuscular diseases. J Neuromuscul Dis.

[24] Weiss RB, Vieland VJ, Dunn DM, Kaminoh Y, Flanigan KM (2018). Long-range genomic regulators of THBS1 and LTBP4 modify disease severity in duchenne muscular dystrophy. Ann Neurol.

[25] Fokkema IFaC, Taschner PEM, Schaafsma GCP, Celli J, Laros JFJ, den Dunnen JT (2011). LOVD v.2.0: the next generation in gene variant databases. Hum Mutat.

[26] Lunter G, Goodson M (2011). Stampy: a statistical algorithm for sensitive and fast mapping of Illumina sequence reads. Genome Res.

[27] Li H, Handsaker B, Wysoker A, Fennell T, Ruan J, Homer N (2009). The sequence alignment/Map format and SAMtools. Bioinformatics.

[28] Wang K, Li M, Hakonarson H (2010). ANNOVAR: functional annotation of genetic variants from high-throughput sequencing data. Nucleic Acids Res.

[29] Taylor J (2005). A tail strength measure for assessing the overall univariate significance in a dataset. Biostatistics.

[30] Wu MC, Lee S, Cai T, Li Y, Boehnke M, Lin X (2011). Rare-variant association testing for sequencing data with the sequence kernel association test. Am J Hum Genet.

[31] Böhringer S (2013). Dynamic parallelization of R functions. R J.

[32] Goeman JJ, van de Geer SA, van Houwelingen HC (2006). Testing against a high dimensional alternative. J R Stat Soc Ser B.

[33] Stöckel D, Kehl T, Trampert P, Schneider L, Backes C, Ludwig N (2016). Multi-omics enrichment analysis using the GeneTrail2 web service. Bioinformatics.

[34] Consortium* TGte. (2013). The genotype-tissue expression (GTEx) project. Nat Genet.

[35] Kotlyar M, Pastrello C, Sheahan N, Jurisica I (2016). Integrated interactions database: tissue-specific view of the human and model organism interactomes. Nucleic Acids Res.

[36] ElSharawy A, Manaster C, Teuber M, Rosenstiel P, Kwiatkowski R, Huse K (2006). SNPSplicer: systematic analysis of SNP-dependent splicing in genotyped cDNAs. Hum Mutat.

[37] Koeks Z, Bladen CL, Salgado as D, van Zwet E, Pogoryelova O, McMacken G (2017). Clinical outcomes in duchenne muscular dystrophy: a study of 5345 patients from the TREAT-NMD DMD global database. J Neuromuscul Dis.

[38] Muntoni F, Domingos J, Manzur AY, Mayhew A, Guglieri M, Sajeev G (2019). Categorising trajectories and individual item changes of the North Star Ambulatory Assessment in patients with Duchenne muscular dystrophy. PLoS One.

[39] Emond MJ, Louie T, Emerson J, Zhao W, Mathias RA, Knowles MR (2012). Exome sequencing of extreme phenotypes identifies DCTN4 as a modifier of chronic Pseudomonas aeruginosa infection in cystic fibrosis. Nat Genet.

[40] Kumar P, Henikoff S, Ng PC (2009). Predicting the effects of coding non-synonymous variants on protein function using the SIFT algorithm. Nat Protoc.

[41] Adzhubei IA, Schmidt S, Peshkin L, Ramensky VE, Gerasimova A, Bork P (2010). A method and server for predicting damaging missense mutations. Nat Methods.

[42] Petryszak Robert, Burdett Tony, Fiorelli Benedetto, Fonseca Nuno A., Gonzalez-Porta Mar, Hastings Emma, Huber Wolfgang, Jupp Simon, Keays Maria, Kryvych Nataliya, McMurry Julie, Marioni John C., Malone James, Megy Karine, Rustici Gabriella, Tang Amy Y., Taubert Jan, Williams Eleanor, Mannion Oliver, Parkinson Helen E., Brazma Alvis (2013). Expression Atlas update—a database of gene and transcript expression from microarray- and sequencing-based functional genomics experiments. Nucleic Acids Research.

[43] Rolland T, Taşan M, Charloteaux B, Pevzner SJ, Zhong Q, Sahni N (2014). A proteome-scale map of the human interactome network. Cell.

[44] Bakay M (2006). Nuclear envelope dystrophies show a transcriptional fingerprint suggesting disruption of Rb-MyoD pathways in muscle regeneration. Brain.

[45] van Setten J, Verweij N, Mbarek H, Niemeijer MN, Trompet S, Arking DE et al. Genome-wide association meta-analysis of 30,000 samples identifies seven novel loci for quantitative ECG traits. Eur J Hum Genet. 2019: 952–62. 10.1038/s41431-018-0295-z PMC6777533 30679814

