# Supplementary material for: TCTEX1D1 is a genetic modifier of disease progression in Duchenne muscular dystrophy
Source: Eur J Hum Genet. 2020 Jan 2;28(6):815–25. doi: 10.1038/s41431-019-0563-6 (PMC7253478; doi:10.1038/s41431-019-0563-6)
Supplement: Supplementary file 1 — Supplementary Table 1 [file 41431_2019_563_MOESM1_ESM.pdf]

HGVS description(s)	rs number	Gene	Study
NC_000001.11:g.7820623T>G	rs10462020	PER3	ELOA vs LLOA
NC_000001.11:g.7837073A>G	rs10462021	PER3	ELOA vs LLOA
NC_000001.11:g.161781951A>G	rs1058405	ATF6	ELOA vs LLOA
NC_000001.11:g.66770414A>T	rs1060575	TCTEX1D1	ECM vs LS
NC_000001.11:g.168044612T>C	rs11558511	DCAF6	ECM vs LS
NC_000001.11:g.179444970A>C	rs11577579	AXDND1	ELOA vs LLOA
NC_000001.11:g.171149701G>A	rs2272797	FMO6P	ECM vs LS
NC_000001.11:g.78891675G>C	rs2275902	ELTD1	ECM vs LS
NC_000001.11:g.36341880C>T	rs3795498	STK40	ELOA vs LLOA
NC_000001.11:g.66776404G>A	rs3816989	TCTEX1D1	ECM vs LS
NC_000001.11:g.207473553T>A	rs4308977	CR2	ELOA vs LLOA
NC_000001.11:g.10954061C>T	rs45537241	C1orf127	ELOA vs LLOA
NC_000001.11:g.10955108A>G	rs75130475	C1orf127	ELOA vs LLOA
NC_000001.11:g.156671745C>T	rs951781	NES	ELOA vs LLOA
NC_000001.11:g.46361784A>G	rs9865	NSUN4	ELOA vs LLOA
NC_000002.12:g.241082327G>C	rs2108485	MTERFD2	ECM vs LS
NC_000002.12:g.135833176C>A	rs3754689	LCT	ELOA vs LLOA
NC_000002.12:g.238131577G>A	rs73102309	ESPNL	ELOA vs LLOA
NC_000003.12:g.108916126C>A	rs10933973	GUCA1C	ELOA vs LLOA
NC_000003.12:g.44651072C>A	rs2272044	ZNF35	ELOA vs LLOA
NC_000003.12:g.195770272C>A	rs2293232	MUC4	ECM vs LS
NC_000003.12:g.19533453A>G	rs33915638	KCNH8	ECM vs LS
NC_000004.12:g.176177134G>A	rs17625943	WDR17	ELOA vs LLOA
NC_000004.12:g.109660207T>A	rs4698744	CCDC109B	ELOA vs LLOA
NC_000004.12:g.6609162G>A	rs61733402	MAN2B2	ECM vs LS
NC_000005.10:g.13788777C>A	rs10513155	DNAH5	ELOA vs LLOA
NC_000005.10:g.127455590G>A	rs17164935	MEGF10	ELOA vs LLOA
NC_000005.10:g.141490949A>G	rs2074912	PCDHGC5	ELOA vs LLOA
NC_000005.10:g.32786283A>C	rs2270915	NPR3	ELOA vs LLOA
NC_000005.10:g.35965868A>C	rs3756669	UGT3A1	ELOA vs LLOA
NC_000005.10:g.7303764G>A	rs6868691	LOC442132	ECM vs LS
NC_000005.10:g.141392860T>A	rs726684	PCDHGA8	ELOA vs LLOA
NC_000006.12:g.75931086G>A	rs10943299	IMPG1	ELOA vs LLOA
NC_000006.12:g.70023655C>G	rs2273426	COL19A1	ECM vs LS
NC_000006.12:g.54940890A>C	rs239798	FAM83B	ECM vs LS
NC_000006.12:g.160531806G>A	rs3124784	LPA	ECM vs LS
NC_000006.12:g.83128954G>A	rs4706980	DOPEY1	ELOA vs LLOA
NC_000006.12:g.42659696G>A	rs6917033	UBR2	ECM vs LS
NC_000006.12:g.149889587C>T	rs6925151	RAET1E-AS1; RAET1E	ECM vs LS
NC_000006.12:g.82191094G>A	rs9449444	IBTK	ELOA vs LLOA
NC_000006.12:g.54941691C>A	rs9475077	FAM83B	ECM vs LS
NC_000007.14:g.102934473G>C	rs1057066	FBXL13; LRRC17	ECM vs LS
NC_000007.14:g.103120064A>C	rs12540583	NAPEPLD	ECM vs LS
NC_000007.14:g.37907501C>T	rs1802074	SFRP4	ECM vs LS
NC_000007.14:g.30452621C>T	rs2075820	NOD1	ELOA vs LLOA
NC_000007.14:g.90372182G>C	rs35001814	GTPBP10	ELOA vs LLOA
NC_000007.14:g.24719199G>C	rs754554	DFNA5	ELOA vs LLOA
NC_000008.11:g.100194163G>A	rs17335870	SPAG1	ELOA vs LLOA
NC_000008.11:g.119832542G>A	rs17818842	TAF2	ELOA vs LLOA

NC_000008.11:g.80821492C>A	rs3907424	ZNF704	ECM vs LS
NC_000009.12:g.104768871G>C	rs10761084	NIPSNAP3B	ECM vs LS
NC_000009.12:g.72700522G>A	rs1796993	TMC1	ELOA vs LLOA
NC_000009.12:g.98985952G>A	rs2075662	COL15A1	ELOA vs LLOA
NC_000009.12:g.21029331T>C	rs2298260	PTPLAD2	ELOA vs LLOA
NC_000009.12:g.104770894C>G	rs3739741	NIPSNAP3B	ECM vs LS
NC_000010.11:g.49325192G>A	rs12217617	C10orf71	ECM vs LS
NC_000010.11:g.43616797G>A	rs12354886	ZNF485	ELOA vs LLOA
NC_000010.11:g.113590838G>A	rs1885434	NRAP	ELOA vs LLOA
NC_000010.11:g.53995731C>T	rs2135720	PCDH15	ELOA vs LLOA
NC_000010.11:g.100296988C>T	rs2278842	PKD2L1	ELOA vs LLOA
NC_000010.11:g.49323927A>C	rs45554335	C10orf71	ECM vs LS
NC_000010.11:g.15607699T>C	rs9333269	ITGA8	ECM vs LS
NC_000011.10:g.58724448A>T	rs10896818	GLYAT	ELOA vs LLOA
NC_000011.10:g.64259167G>A	rs12146487	PLCB3	ECM vs LS
NC_000011.10:g.36575763A>G	rs2227973	RAG1	ELOA vs LLOA
NC_000011.10:g.5507922A>G	rs2234455	UBQLN3	ELOA vs LLOA
NC_000011.10:g.118198037T>C	rs2298831	AMICA1	ELOA vs LLOA
NC_000011.10:g.1083116G>A	rs41345745	MUC2	ECM vs LS
NC_000011.10:g.1083761C>G	rs57737240	MUC2	ECM vs LS
NC_000011.10:g.18934649C>T	rs78179510	MRGPRX1	ECM vs LS
NC_000012.12:g.124968569C>T	rs11057939	DHX37	ELOA vs LLOA
NC_000012.12:g.66331422C>T	rs1168312	HELB	ELOA vs LLOA
NC_000012.12:g.14806457T>C	rs2241221	C12orf69	ELOA vs LLOA
NC_000012.12:g.14882147T>A	rs4236	MGP	ECM vs LS
NC_000012.12:g.52424593C>G	rs61730614	KRT75	ELOA vs LLOA
NC_000012.12:g.133191967A>G	rs61960670	ZNF268	ELOA vs LLOA
NC_000012.12:g.14823129A>C	rs7304054	C12orf60	ELOA vs LLOA
NC_000012.12:g.7741460G>A	rs73056605	CLEC4C	ELOA vs LLOA
NC_000012.12:g.14823244T>A	rs7307438	C12orf60	ELOA vs LLOA
NC_000013.11:g.44574121G>A	rs9525983	TSC22D1-AS1; TSC22D1	ELOA vs LLOA
NC_000013.11:g.23593366T>A	rs9550987	TNFRSF19	ECM vs LS
NC_000014.9:g.95544087G>A	rs11628901	GLRX5	ELOA vs LLOA
NC_000014.9:g.24118430G>A	rs3825584	DCAF11	ECM vs LS
NC_000015.10:g.82759440C>T	rs1108134	FSD2	ECM vs LS
NC_000015.10:g.83011577G>A	rs17361375	C15orf40	ECM vs LS
NC_000015.10:g.90257217C>T	rs2063743	TTLL13	ELOA vs LLOA
NC_000015.10:g.74825571G>T	rs79217743	CPLX3; LMAN1L	ECM vs LS
NC_000016.10:g.3657746G>A	rs1053874	DNASE1	ELOA vs LLOA
NC_000016.10:g.1220111G>A	rs1054644	CACNA1H	ECM vs LS
NC_000016.10:g.3317229T>C	rs17611866	ZNF75A	ECM vs LS
NC_000016.10:g.3289435G>A	rs220379	ZNF263	ECM vs LS
NC_000016.10:g.71284674T>C	rs3803704	FTSJD1	ELOA vs LLOA
NC_000016.10:g.20465530T>C	rs7187246	ACSM2A	ECM vs LS
NC_000016.10:g.81123780G>T	rs8050204	PKD1L2	ELOA vs LLOA
NC_000017.11:g.15607574G>A	rs62070401	CDRT1	ELOA vs LLOA
NC_000017.11:g.40799739C>A	rs7209228	KRT28	ELOA vs LLOA
NC_000017.11:g.40958710C>T	rs7213256	KRT39	ELOA vs LLOA
NC_000017.11:g.68271550A>T	rs7222013	ARSG; SLC16A6	ELOA vs LLOA
NC_000018.10:g.6985271C>G	rs11664063	LAMA1	ELOA vs LLOA

NC_000018.10:g.2595444G>T	rs12456560	NDC80	ECM vs LS
NC_000018.10:g.7034509T>G	rs566655	LAMA1	ELOA vs LLOA
NC_000018.10:g.6993674T>C	rs62081533	LAMA1	ELOA vs LLOA
NC_000019.10:g.8371280C>T	rs1044250	ANGPTL4	ECM vs LS
NC_000019.10:g.18386331T>A	rs1059369	GDF15	ELOA vs LLOA
NC_000019.10:g.8056476A>G	rs2032887	CCL25	ELOA vs LLOA
NC_000019.10:g.18218974C>T	rs2229228	PDE4C	ECM vs LS
NC_000019.10:g.18265036G>A	rs2277921	KIAA1683	ELOA vs LLOA
NC_000019.10:g.35121255C>G	rs2290649	FXYD3	ELOA vs LLOA
NC_000019.10:g.8103661C>T	rs33967815	FBN3	ELOA vs LLOA
NC_000019.10:g.11214289C>T	rs34243815	DOCK6	ECM vs LS
NC_000019.10:g.18765499A>G	rs3746266	CRTC1	ECM vs LS
NC_000019.10:g.35812762G>A	rs3848666	PRODH2	ECM vs LS
NC_000019.10:g.48613102T>C	rs447802	FAM83E	ECM vs LS
NC_000019.10:g.17543372C>T	rs45532635	FAM129C	ECM vs LS
NC_000019.10:g.46494979C>T	rs4803967	PNMAL2	ELOA vs LLOA
NC_000019.10:g.10003576C>A	rs61742765	COL5A3	ELOA vs LLOA
NC_000019.10:g.37885026C>T	rs73027451	WDR87	ECM vs LS
NC_000020.11:g.35286917C>T	rs2425049	FAM83C	ECM vs LS
NC_000020.11:g.56248973G>A	rs3827103	MC3R	ELOA vs LLOA
NC_000022.11:g.29512083C>T	rs1049534	THOC5	ELOA vs LLOA
NC_000022.11:g.50077414C>A	rs6010260	MLC1	ELOA vs LLOA
