# Supplementary figures and images for: TCTEX1D1 is a genetic modifier of disease progression in Duchenne muscular dystrophy
Source: Eur J Hum Genet. 2020 Jan 2;28(6):815–25. doi: 10.1038/s41431-019-0563-6 (PMC7253478; doi:10.1038/s41431-019-0563-6)

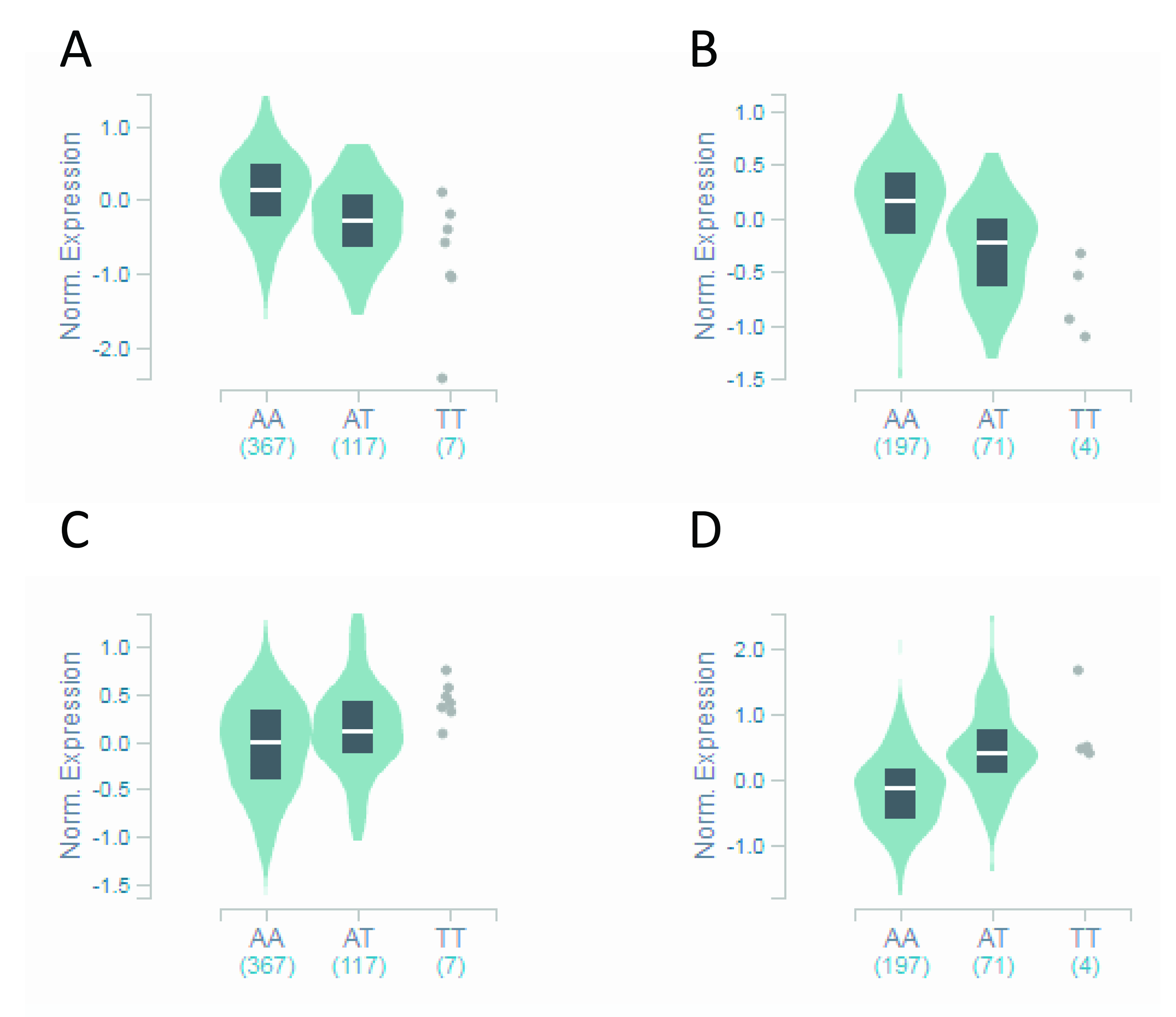

Supplement: Supplementary file 2 — Supplementary Figure 1 [file 41431_2019_563_MOESM2_ESM.tif]
