# Supplementary material for: TCTEX1D1 is a genetic modifier of disease progression in Duchenne muscular dystrophy
Source: Eur J Hum Genet. 2020 Jan 2;28(6):815–25. doi: 10.1038/s41431-019-0563-6 (PMC7253478; doi:10.1038/s41431-019-0563-6)
Supplement: Supplementary file 3 — Supplemental Material Legends [file 41431_2019_563_MOESM3_ESM.docx]

**Supplemental Material Legends**

**Supplementary Figure 1.** Violin plots showing the expression quantitative trait loci (eQTL) associations in muscle and heart for SNP rs1060575 (data obtained from GTEx database). AB. Violin plots showing *TCTEX1D1* gene expression across the possible genotypes in muscle (A) and left ventricle (B) tissue. C-D. Violin plots showing *SGIP1* gene expression across genotypes in muscle (C) and the left ventricle (D) tissue. All associations are highly significant (*P*<0.0001).

**Supplementary Table 1.** List of the 121 variants included in the analysis of the BIO-NMD cohort. Variant description is provided according to HGVS guidelines. SNP rs number is provided as well as the gene name and the discovery study in which the variant was identified.
